# SERS biosensor with plastic antibodies for detection of a cancer biomarker protein

**DOI:** 10.1007/s00604-024-06327-y

**Published:** 2024-04-04

**Authors:** Daniela Oliveira, Mariana C. C. G. Carneiro, Felismina T. C. Moreira

**Affiliations:** CIETI - LabRISE-School of Engineering, Polytechnic of Porto, R. Dr. António Bernardino de Almeida, 431, 4249-015 Porto, Portugal

**Keywords:** Molecular imprinting, Surface-enhanced Raman scattering, Cancer biomarkers, Biosensor

## Abstract

Surface-enhanced Raman scattering (SERS) is a powerful method for detecting breast cancer-specific biomarkers due to its extraordinary enhancement effects obtained by localized surface plasmon resonance (LSPR) in metallic nanostructures at hotspots. In this research, gold nanostars (AuNSs) were used as SERS probes to detect a cancer biomarker at very low concentrations. To this end, we combined molecularly imprinted polymers (MIPs) as a detection layer with SERS for the detection of the biomarker CA 15–3 in point-of-care (PoC) analysis. This required two main steps: (i) the deposition of MIPs on a gold electrode, followed by a second step (ii) antibody binding with AuNSs containing a suitable Raman reporter to enhance Raman signaling (SERS). The MPan sensor was prepared by electropolymerization of the monomer aniline in the presence of CA 15–3. The template molecule was then extracted from the polymer using sodium dodecyl sulfate (SDS). In parallel, a control material was prepared in the absence of the protein (NPan). Surface modification for the control was performed using electrochemical techniques such as cyclic voltammetry (CV) and electrochemical impedance spectroscopy (EIS). The performance of the sensor was evaluated using the SERS technique, in which the MPan sensor is first incubated with the protein and then exposed to the SERS probe. Under optimized conditions, the device showed a linear response to CA 15–3 concentrations from 0.016 to 248.51 U mL^−1^ in a PBS buffer at pH 7.4 in 1000-fold diluted serum. Overall, this approach demonstrates the potential of SERS as an optical reader and opens a new avenue for biosensing applications.

**Graphical Abstract:**

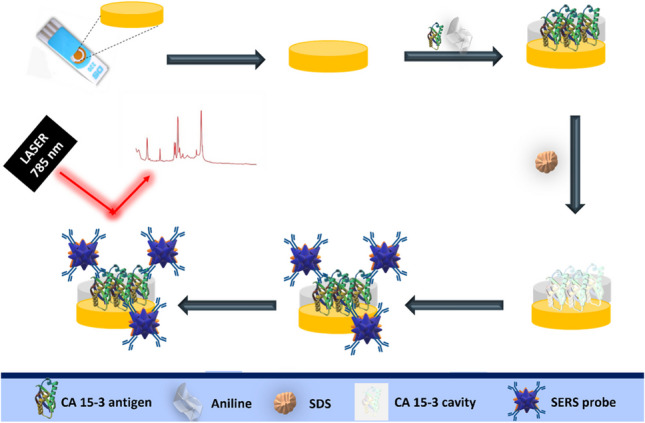

**Supplementary Information:**

The online version contains supplementary material available at 10.1007/s00604-024-06327-y.

## Introduction

Surface-enhanced Raman scattering (SERS) offers great advantages in biosensing applications, including exceptional sensitivity for single molecule detection, multiplexing capabilities, label-free detection with high chemical specificity by Raman spectroscopy, and minimal sample preparation [1, 2]. In SERS techniques, various nanostructures, such as gold nanoparticles (AuNPs), silver, and carbon nanotubes, are used to amplify the Raman signal and increase sensitivity [3, 4]. The selection depends on the properties of the analyte and the requirements of the application. AuNPs offer advantages in SERS, including strong plasmonic properties, conformability, biocompatibility, stability, and ease of functionalisation for selective detection. Gold nanostars (AuNSs) are a promising subtype of gold nanostructures as they improve SERS signaling due to their branched structure, large surface area, and versatility [5, 6].

The growing world population and rising disease rates have created a need for user-friendly, reliable, fast, and cost-effective point-of-care (PoC) devices. SERS has been used extensively to diagnose disease as it can detect extremely small amounts with subpicomolar sensitivity [7]. The growing popularity of SERS in biosensing is due to its seamless integration into liquid samples due to minimal water scattering [8, 9]. In contrast to conventional tests, SERS enables measurements in liquids, gases, solids, and powders, providing specific molecular insights and often revealing a distinctive vibrational fingerprint of the molecules or cells under examination [10]. SERS has been used for the detection of conditions such as Alzheimer’s [11–13], diabetes [14], inflammation [15], Crohn’s disease [16], and single Hb molecule [17], to name a few.

Breast cancer is the second most common cause of death in women after lung cancer [18, 19]. The diagnosis is usually made by mammography, ultrasound, magnetic resonance imaging (MRI), or biopsy. In addition, it is often confirmed by histopathological examination, which is unfortunately time-consuming and highly prone to human interpretation errors [19]. Nowadays, biosensors are a promising tool that allows use at the PoC due to its portability, which limits the implementation of comprehensive screening programs for early detection. Therefore, researchers are looking for new approaches that can be used at the PoC [20, 21]. Various biosensors have been described in the literature for the detection of the biomarker CA 15–3 using antibodies or molecularly imprinted polymers (MIPs) as a biological recognition layer (Table S1). However, to our knowledge, this study is the first to report the integration of MIPs and SERS for the detection of the CA 15–3 protein. In SERS-based detection, metal NPs are coated with an antibody and a Raman reporter (SERS tag) in a sandwich immunoassay [3, 22]. Specific antibodies on SERS substrates indirectly determine the amount of antigen by measuring the SERS signal intensity [23]. Several studies are exploring SERS for the detection of cancer biomarkers, such as CA 15–3 (MUC1) [19]. Gold nanorods (AuNRs) with silver nanoparticles (AgNPs) were developed for the highly sensitive detection of MUC1 at 4.3 amol L^−1^. The process involved the formation of AgNP and AuNR core-satellite nanostructures that generated high SERS signals through MUC1-specific target-DNA coupling. Specific recognition resolved the core-satellite junctions and abolished the SERS signal [24]. Further improvement of MUC1 detection was achieved by magnetic separation, SERS, and colorimetric visualization, resulting in a detection limit of 0.1 U mL^−1^ [25]. The proposed biosensor utilized magnetic nanobeads with MUC1-specific aptamers as capture probes and Raman reporters modified with gold-silver core–shell nanoparticles and complementary MUC1 sequences as signal indicators. This innovative strategy proved to be effective for the ultrasensitive detection of MUC1 in real samples.

Although SERS is a very powerful tool, there are problems with selectivity, reproducibility, and stability. To solve these problems, MIPs that mimic natural receptor-ligand interactions are used. MIPs provide specific binding sites that correspond to the shape, size, and functional groups of the template molecule [26–29]. Due to their binding sites, these biomimetic polymers have a capture selectivity that enables recognition similar to that of natural antibodies, but with greater structural stability [30]. The MIP-based SERS biosensor has a “memory” function that improves selectivity and performance. In a 2019 study, Carneiro et al. demonstrated dual bio-recognition by combining MIP and antibody in SERS detection of carcinoembryonic antigen (CEA). The biosensor detected the biomarker in the range of ng mL^−1^, with no significant difference in detection limits between MIP with electrochemical and optical transduction [31].

In this research, the MIP-SERS sensor was prepared by the electropolymerization (ELP) of polyaniline (Pan) onto the gold working electrode of a commercial gold screen-printed electrode (Au-SPE). To obtain a sandwich assay, antibodies were immobilized on AuNSs loaded with 4-aminothiophenol (4-ATP), which served as a Raman reporter molecule. Overall, this innovative MIP-SERS sensor platform offers a non-destructive, fast, user-friendly, and quantitative test method with promising applications in clinical diagnosis and prognosis.

## Experimental section

### Instrumentation

Electrochemical measurements were performed in a Metrohm Autolab potentiostat/galvanostat equipped with an FRA module and controlled by Nova 2.1.6 software. The Au-SPEs (DRP-220AT, DropSens) contained a gold working electrode (4 mm), a gold counter electrode, and a pseudo-reference electrode with silver electrical contacts. The switch box connecting these Au-SPEs to the potentiostat was obtained from DropSens. Raman studies were performed with a Thermo Fisher Scientific Company DXR Raman spectrometer using Thermo Scientific OMNIC software. Spectra were recorded in the range of 300 to 1800 using a 785-nm excitation laser through a 50 × confocal microscope objective. Laser power was set at 10 mW, with an aperture of 50 µm slit, for an acquisition time of 10 seconds. UV–Vis studies were performed using the Evolution 220 UV–Vis spectrophotometer from Thermo Fisher Scientific Company. Transmission electron microscopy (TEM) was performed using a JEOL JEM 1010 transmission electron microscope operating at an accelerating voltage of 100 kV. SEM was performed using a JEOL JSM 6301 F/Oxford INCAEnergy 350/Gatan Alto 2500 high-resolution field emission scanning electron microscope.

### Reagents

All chemicals were of analytical grade and the water was ultra-pure Milli-Q laboratory water. Potassium ferricyanide II-3-hydrate (K_4_Fe(CN)_6_∙3H_2_O) and potassium ferricyanide III (K_3_Fe(CN)_6_) were purchased from Riedel de Haën; N,N-dimethylformamide (DMF) (C_3_H7NO), tri-sodium citrate dihydrate (C_6_H_5_Na_3_O_7∙_2H_2_O), and aniline (C_6_H_7_N) were purchased from Analar Normapur; ethanol absolute (C_2_H_5_OH) ≥ 99.9%, hydrotetrachloric acid (HAuCl4∙3H_2_O), trypsin, and polyvinylpyrrolidone (PVP, (C_6_H_9_NO)n) with a molecular weight of 10,000 were purchased from Sigma-Aldrich; 4-ATP (C_6_H_7_NS) ≥ 97% was purchased from Merck; glucose was purchased from Alfa Aesar; hydrochloric acid (HCl) 37%, nitric acid (HNO_3_) 70%, and phosphate-buffered saline (PBS, 0.01 M, pH 7.4) were purchased from Panreac; sulfuric acid (H_2_SO_4_) was purchased from BDH; SDS was purchased from TCI; CORMAY serum HN was purchased from Cormay®; carcinoembryonic antigen was purchased from EastCostBio; cancer antigen 125 (CA-125) was purchased from Hytest; CA 15–3 from human host (reference MBS536585) was purchased from MyBioSource; CA 15–3 antibody (mucin 1 monoclonal antibody Vu-2G7, reference SC-69644) was purchased from Santa Cruz Biotechnology.

### Electrochemical and optical procedures

Electrochemical assays were performed indirectly using 5.0 mmol L^−1^ K_3_[Fe(CN)_6_] and 5.0 mmol L^−1^ K_4_[Fe(CN)_6_] as redox probes in PBS buffer. Electrochemical impedance spectroscopy (EIS) and cyclic voltammetry (CV) were used to characterize the sensors in the different steps of biosensor assembly. EIS assays were also performed in triplicate with the same redox couple [Fe(CN)6]^3−/4−^ at a standard potential of + 0.12 V, using a sinusoidal potential perturbation with an amplitude of 0.01 V and multiple frequencies equal to 50, logarithmically distributed over a frequency range of 0.1–100 kHz. For the calibration curves, CA 15–3 standard solutions in the range of 0.15680 U mL^−1^ and 15,680.0 U mL^−1^ were used, prepared in PBS buffer (pH 7.4).

For the calibration curves of SERS, CA 15–3 standard solutions in the range of 0.016 U mL^−1^ and 248.5 U mL^−1^ prepared in PBS buffer (pH 7.4) and Cormay serum diluted 1:1000 in PBS buffer (pH 7.4) were used. Each solution was allowed to stand on the electrode surface for 20 min. Selectivity studies were performed using a competitive assay in which CA 15–3 (30 U mL^−1^) was mixed with CEA (2.5 ng mL^−1^), CA 125 (35 U mL^−1^), and glucose (0.7 mg mL^−1^). All these solutions were prepared in triplicate in PBS buffer with a pH of 7.4.

### Assembly of the plastic antibody on Au-SPE

**Fig. 1:**
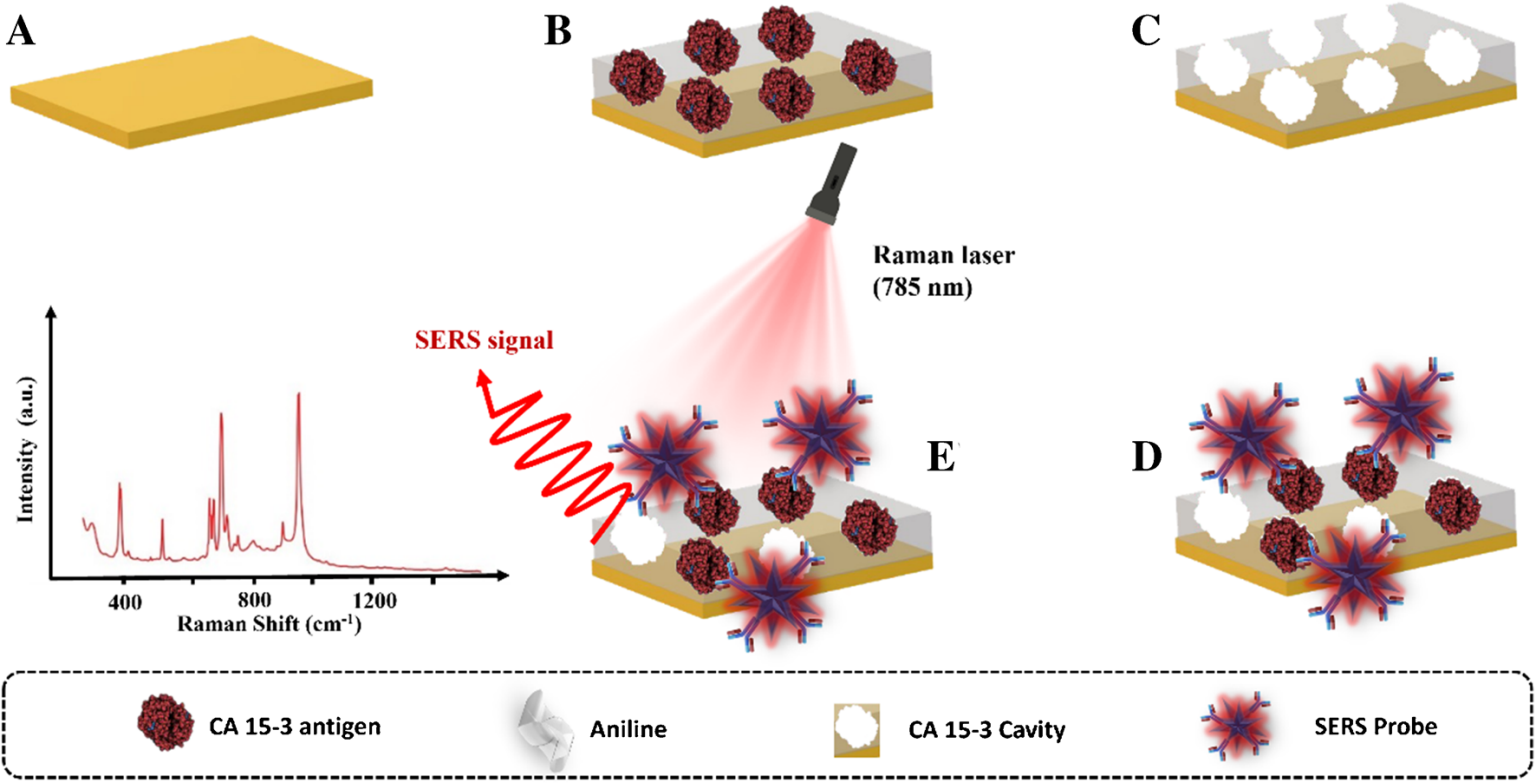
Schematic representation of the assembly of the biosensor and SERS probe for the detection of the target protein CA 15–3. **A** Electrochemical cleaning (H_2_SO_4_). **B** ELP of the monomer with the target molecule. **C** Binding site formation by extraction of the target molecule with SDS. **D** Rebinding of protein and bound of the SERS probe. **E** Signal measurement

The MIP film was applied to the working electrode region of the Au-SPEs as shown in Fig. 1. First, the Au-SPEs (Fig. 1A) were electrochemically cleaned, and CV purified with H_2_SO_4_, 0.5 mol L^−1^. CV scans were performed in the potential range of − 0.2 V to + 1.2 V at a scan rate of 0.05 V s^−1^ for five cycles. This step is extremely important as it promotes activation and homogenization of the working range of the different electrodes. Next, 50 µL of the polymerization solution containing 10 mmol L^−1^ aniline monomer and 700 U mL^−1^ CA 15–3 (as the target molecule) prepared in PBS buffer at pH 7.4 was added to the pretreated surface of the working electrode (WE) (Fig. 1B). ELP was then performed by CV in a potential range between − 0.2 and + 0.9 V with a potential sweep rate of 50 mV s^−1^ for five consecutive cycles (Figure S2). Finally, the target molecule was removed by incubating 5 µL of a solution containing 10% SDS overnight at room temperature on WE (RT) (Fig. 1C).

Non-imprinted polymers (NIPs) were also produced using the same protocol without adding the target molecule to the ELP solution. The NIPs are used as a control in protein recognition.

### Synthesis of the gold nanostars (AuNSs)

The AuNSs were obtained using gold seeds, so we initially synthesized the gold nanoparticles (AuNPs) previously described with minor modifications by Dong et al. [32]. AuNPs were prepared from a 0.5 mM HAuCl_4_ aqueous solution, which was boiled with vigorous stirring, and 5 mL of tri-sodium citrate (34 mmol L^−1^) solution was added. The reaction was allowed to run at the same temperature for 20 min, and then, the resulting solution was cooled. Then, the surface of AuNPs was coated by gradually adding a 5 mL solution of 2.5 mmol L^−1^ PVP and stirring for 24 h, forming the gold seeds. In the following phase, gold seeds were centrifuged (4500 rpm, 45 min) to remove excess PVP. The seeds were then re-suspended in ethanol. The absorbance was measured at this time to check the concentration of the sample and adjusted to 1.5 mmol L^−1^ (Figure S1 A). The concentration calculation was performed using the UV–vis absorbance method recommended by Scarabelli et al. [33].

A solution of PVP in DMF (0.9 mol L^−1^, 15 mL) was then prepared and added briskly to a HAuCl_4_ solution (105.6 mmol L^−1^), stirring for 5 min. Then, 320 µL of the gold seeds was incorporated and the mixture was stirred in an ice bath for 60 min. Again, the centrifugation steps were repeated to remove the DMF, and the resulting sediment (AuNSs) was resuspended in ethanol. The final concentration of AuNSs (Figure S1 B) was determined using the UV–Vis absorption method recommended by Scarabelli et al. [33, 34].

### SERS probe assembly

The SERS probe (AuNSs/4-ATP/Ab-CA 15–3) was made according to the procedure described by Carneiro et al. [35]. First, a mixture containing 0.01 mmol L^−1^ 4-ATP (dissolved in pure ethanol) and 0.6 mmol L^−1^ AuNSs was prepared in equal parts. This mixture was shaken for 2 h at a temperature of 25 °C. Then, the resulting solution was centrifuged at 4500 rpm for 20 min at a temperature of 25 °C to remove excess unbound 4-ATP. The resulting sediment (AuNSs/4-ATP) was again suspended in 95 µL PBS buffer at pH 7.4. Subsequently, 5 µL Ab-CA 15–3 (200 µg mL^-1^) was added and the resulting solution was stirred for 90 min at RT to allow the formation of AuNSs/4-ATP/Ab-CA 15–3. The resulting mixture was subjected to the same centrifugation procedure as mentioned previously to remove unbound Ab-CA 15–3. The resulting pellet was resuspended in 200 µL PBS, giving the final solution of the SERS probe.

### SERS CA 15–3 detection

Detection of CA 15–3 began with incubation of a CA 15–3 standard solution at increasing concentrations (0.016 and 248.5 U mL^−1^) on the working electrode surface (where the MPan film was mounted) for a period of 20 min (Fig. 1D). The surface was then washed and 5 µL of the SERS probes (AuNSs/4-ATP/Ab-CA 15–3) was incubated overnight at the same location (Fig. 1D). Raman spectra were then recorded (Fig. 1E).

## Results and discussion

### Electrochemical characterization

The biorecognition element of the biosensor was assembled on an Au-SPE pretreated with sulfuric acid by CV. This pretreatment is responsible for activating the electrode and oxidizing the impurities present on the surface. These supports were then modified with the so-called plastic antibody prepared on-site by ELP of aniline in the presence of the target compound, CA 15–3 protein (Fig. 1B).

The selection of best conditions for aniline ELP in terms of potential range and scan rate was based on previous work of the authors of this research work (− 0.2 to + 0.9 V) and (50 mV s^−1^) [1, 2]. The number of cycles of ELP was optimized (5, 10, and 20, respectively) for polymers without the template molecule (NIPs). Figure S3 shows the CV spectra of ELP for each number of cycles. Figure S4 shows the CV and EIS spectra for the different numbers of cycles analyzed. Figure S4, the first columns show the CV and EIS spectra of a clean gold with low peak separation in CV and low charge resistance in EIS, which is expected if the bare gold normally has a very fast electron transfer. After ELP of aniline, a decrease in current and peaks in the voltammograms (Figure S4, first row) and an increase in charge transfer resistance in impedance measurements (Figure S4, second row) were observed for each cycling condition. These changes are remarkable considering that the polymer resulting from this process exhibits insulating behavior in all cases. The selection of the best condition was based on the thickness of the film formed and the stability after several measurements. This choice was also influenced by the fact that thicker films can present additional challenges, especially in applications involving the removal of the target molecule. This is especially true for polymers with molecular imprinting, where the film thickness must be carefully considered to ensure an efficient response in the selective removal of the specific molecule. The best condition in terms of electron transfer and stability of the polymer surface was five cycles and was considered for further experiments (Figure S2).

**Fig. 2:**
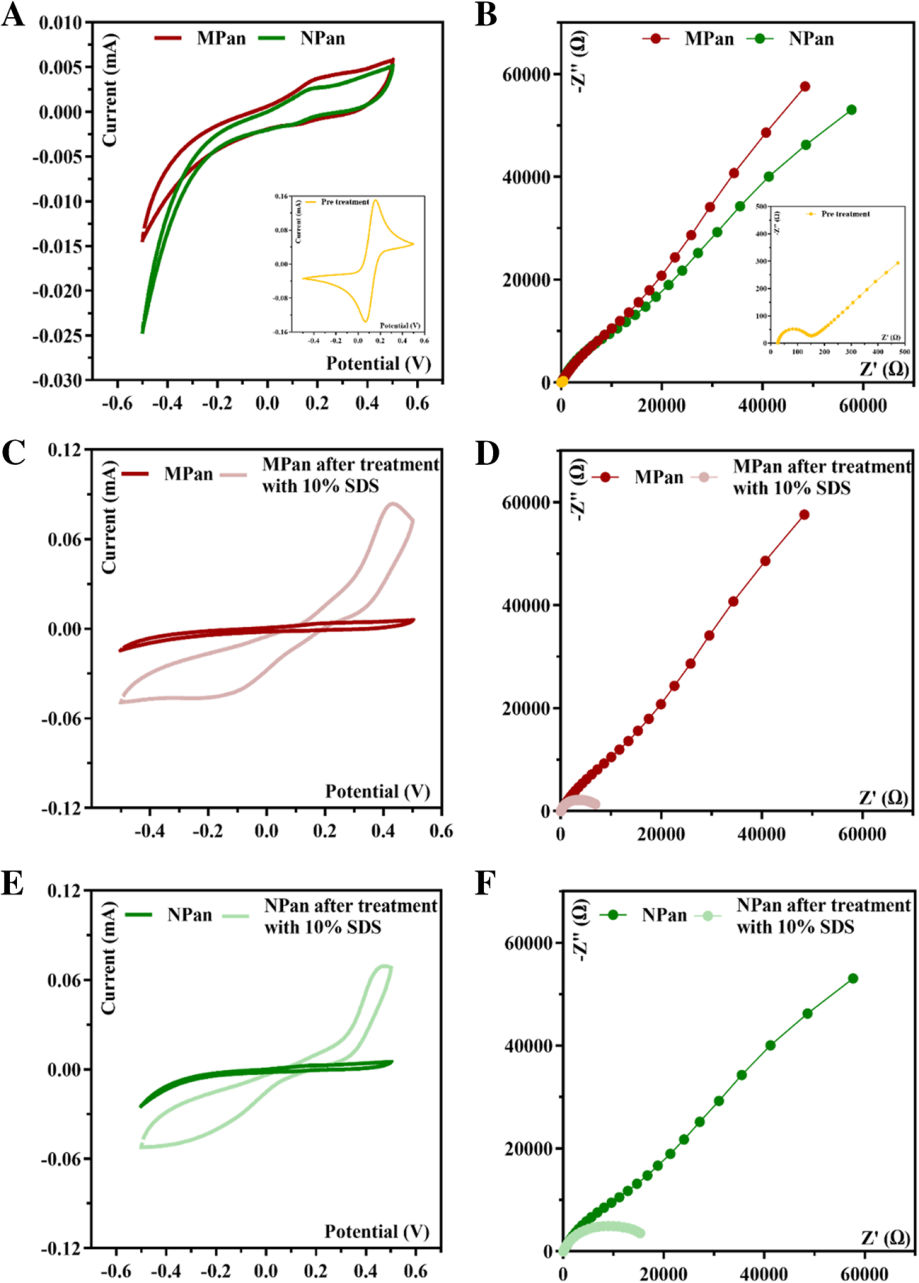
CV and EIS measurements were performed at various stages during the fabrication of MPan and NPan. **A** and **B** The polymerization process for MPan and NPan, respectively. The sensor’s response after the removal of the target molecule is illustrated in **C** and **D** for MPan and **E** and **F** for NPan

The chemical recognition of the protein was combined with the advantages of the biocompatible properties of the polymeric matrix (Pan). The use of aniline enabled (i) ELP and (ii) the establishment of hydrogen bonds or ionic interactions with the protein via the amine residues. In addition, previous studies have shown that both the conductive and non-conductive forms of Pan did not induce inflammatory responses (rodent model) in vivo, indicating good biocompatibility and histocompatibility [36]. This study also showed that the Pan polymer is a suitable matrix for the entrapment of proteins preserving their 3D structure. The surface modification was followed by CV (Fig. 2A) and EIS measurements (Fig. 2B). CV measurements showed an irreversible system for MPan material, without relevant peaks. From Figs. 2A and C, the current of the MPan material with CA 15–3 confined in the polymer matrix is very similar to that of the NPan material. This could be related to the high insulating properties of the polymer at physiological pH. The final step was to remove the protein from the polymer.

In this study, various methodologies for removing the template from the polymer matrix were investigated. Firstly, a 100-fold diluted trypsin solution was applied, and the electrodes were incubated at 37 °C for 2 h. However, this resulted in a significant increase in charge transfer resistance (*R*_CT_) and a slight decrease in current compared to the previous step. These results indicate an absorption of trypsin on the surface of the polymer matrix, which is evident for both MPan and NPan, as shown in Figure S5 (A-B) and Figure S5 (C-D), respectively. To eliminate trypsin and the target molecule, a 10% SDS solution was applied to the working electrode overnight. This phase led to a sharp decrease in *R*_CT_ and an increase in current, indicating the formation of cavities within the imprinted structure, reducing the insulating properties of the original polymer film. After the removal of the target molecule, the electrode surface was stabilized by successive incubations with PBS buffer. Stabilization was observed after four iterations, indicating the development of a uniform and functional surface. Subsequently, the performance of the sensor was evaluated by adding CA 15–3 in increasing concentrations. The results, shown in Figure S6 (A-B) for MIP and Figure S6 (C-D) for NPan, show similar trends in both cases, with minimal differences observed across the range of concentrations tested. This is attributed to the high charge resistance possibly leading to lower sensitivity at lower CA 15–3 concentrations.

Therefore, the strategic decision was made to use only the SDS solution for template removal. This decision was motivated by the need to preserve the sensitivity of the sensor at lower concentrations and to prevent potential saturation of the sensor response due to increased charge resistance in EIS. After this treatment, an increase in the redox peak currents was observed for both MPan and NPan. However, this increase is higher for the MPan than for the NPan material. This behavior is due to the absence of the target molecule.

The EIS measurements are consistent with the CV data. After electrode MPan and NPan polymerization on the Au-SPE surface, a huge increase in the impedance was observed, related to the presence of a great insulating polymer (Fig. 2B). After the removal of the template, a decrease in the electron charge resistance was observed, in the MPan sensor which can be attributed to the presence of cavities. Overall, these results confirm the electrochemical modification of the electrodes.

### SERS probe characterization

**Fig. 3:**
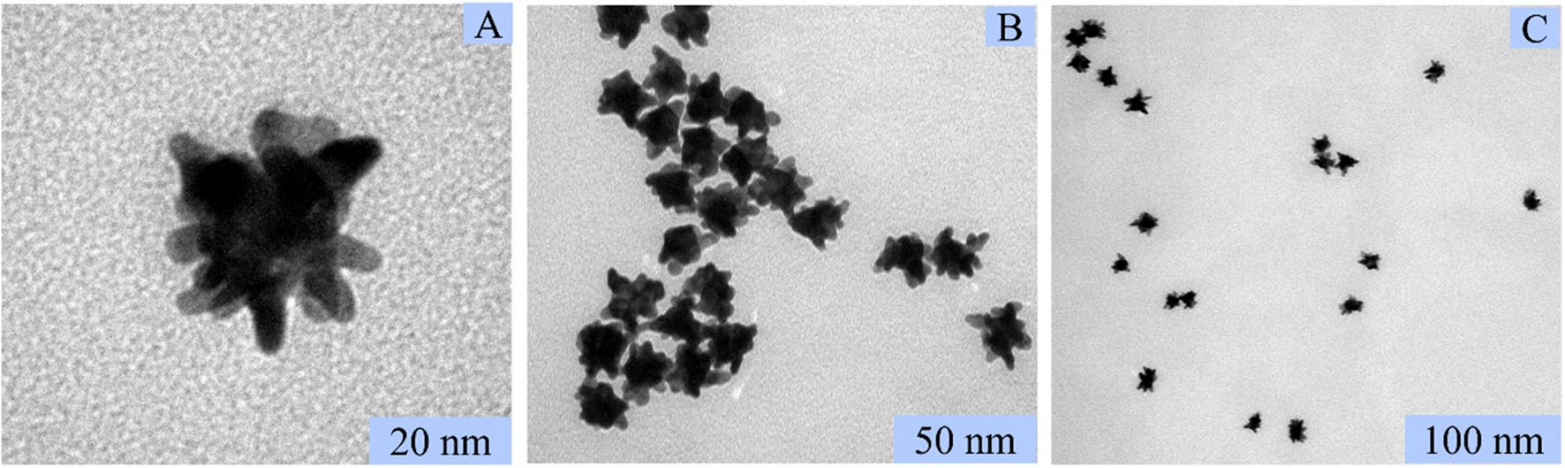
TEM images of the AuNSs at low magnification (**A**, **B**) and high magnification (**C**)

AuNSs obtained here were characterized by TEM analysis. TEM images of isolated AuNSs showed a solid core bounded by several sharp, asymmetric, and short branches (Fig. 3A). In addition, images of multiple AuNSs showed that the particles were of similar size and were individually dispersed rather than aggregated (Fig. 3B). Overall, the TEM images indicate that the average diameter of AuNSs is ~ 30 nm.

**Fig. 4:**
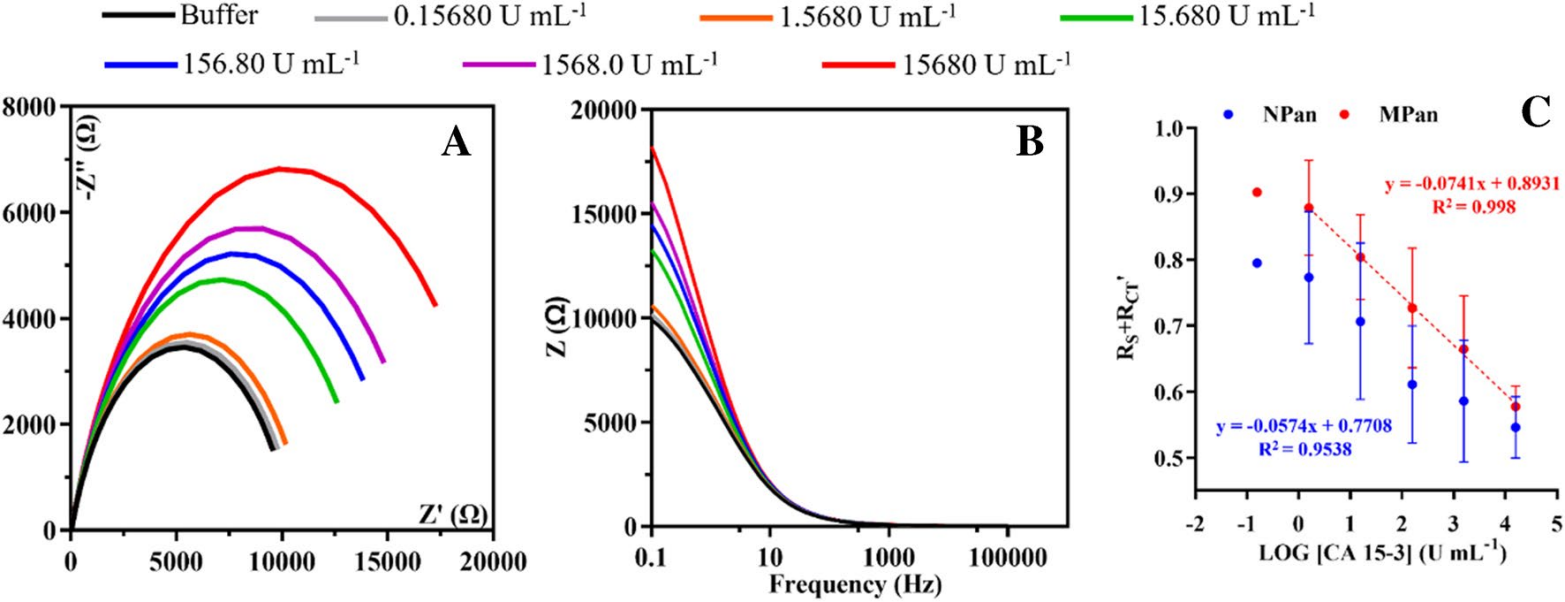
EIS (Nyquist and Bode) measurements of the last stabilization in buffer and increasing standard concentrations of CA 15–3 in MPan (**A** and **B**). The calibration curve for the *R*_s_ + *R*_CT_ response against the logarithmic concentration of CA 15–3 for both MPan and NPan (**C**)

### Analytical response of the electrochemical biosensor

Figure 4A shows the typical Nyquist plots of the biosensor against increasing CA 15–3 concentrations in PBS buffer, pH 7.4; Fig. 4B represents the Bode circuit; and Fig. 4C shows the corresponding EIS calibration curve plotting *R*_CT_ against the logarithm of CA 15–3 concentration. Regarding the performance of NPan (Figure S7), it should be noted that there is also an increase in the signal as a function of increasing concentrations of CA 15–3. The response of the CA 15–3 biosensor was tested under physiological pH conditions. The calibration curve was established by incubating 5 µL in the working area of the electrode with CA 15–3 standard solutions of increasing concentrations, from 0.158 to 1580 U mL^−1^, prepared in PBS buffer pH 7.4. Each standard solution was incubated on the sensor surface for 20 min and then replaced by the hexacyanoferrate redox probe to check the EIS response. The representative data obtained are shown in Fig. 4.

Overall, the best calibration performance was obtained for the MPan sensor, with an average slope of − 0.0741, a squared correlation coefficient *R*^2^ of 0.99, a lower limit of the linear range (LLLR) of 1.568 U mL^−1^, and a limit of detection (LOD) of 0.891 U mL^−1^. In contrast, the calibrations with NPan (Fig. 4C) do not show a linear response, as the squared correlation coefficient was 0.9538.

### Analytical performance in SERS

#### Detection of CA 15–3 in buffer

**Fig. 5:**
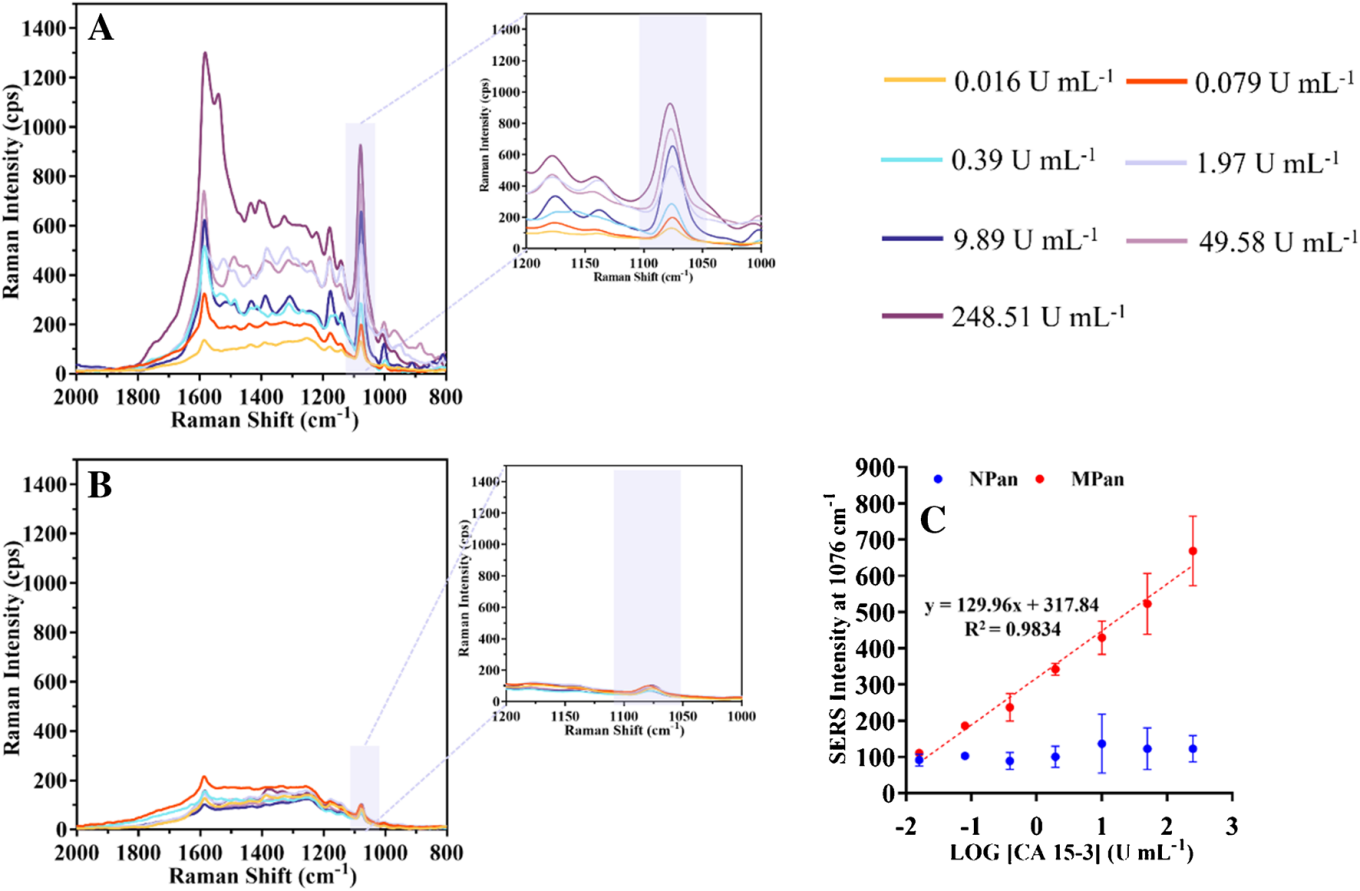
The MPan (**A**) Raman spectra and the NPan (**B**) Raman spectra after incubation in the WE with increasing concentrations of CA 15–3 in a buffer solution (ranging from 0.016 to 248.51 U mL^*−*^^1^), then incubated with a SERS probe. The linear response of the sensor calibration (**C**)

Figure 5A shows the typical RAMAN spectra of the MPan and (Fig. 5B) NPan against increasing CA 15–3 concentrations prepared in PBS buffer, pH 7.4. Figure 5C shows the corresponding SERS signal calibration curve, plotting the SERS signal against the logarithm of CA 15–3 concentration. The calibration curve was performed by incubating 5 µL in the working area of the electrode with CA 15–3 standard solutions of increasing concentrations, from 0.016 to 248.51 U mL^−1^, prepared in PBS buffer pH 7.4.

The MPan showed a lower limit of the linear range of 0.016 U mL^−1^, a linear range within 0.016 to 248.51, and a coefficient of correlation > 0.9834. The peak intensity at 1079 cm^−1^ increased with increasing protein concentrations, demonstrating that a higher number of reporter probes were present. The NPan did not display linear and a very weak signal in all concentration ranges of the work.

Comparing the analytical performance of the SERS-based biosensor for MPan with electrochemical transduction, it becomes evident that the SERS approach exhibits a significant (LLLR), approximately 10 times lower, and a substantially steeper slope. This compelling data unequivocally demonstrates that this innovative approach yields superior results in terms of analytical features. In summary, the results obtained firmly establish that SERS detection significantly enhances the operational characteristics of the biosensor when contrasted with the EIS technique.

#### Selectivity study

The study of the effects of specific compounds present in physiological fluids on the sensor surface is essential for effective analytical application. This was evaluated by examining the response of SERS to interfering species. Glucose, normal composition of serum samples, and presence of some cancer biomarkers such as CA 125 were selected as interfering species. In this study, the mixed solution method was used, in which the target analyte 30 U mL^−1^ was mixed with each biological preparation.

**Fig. 6:**
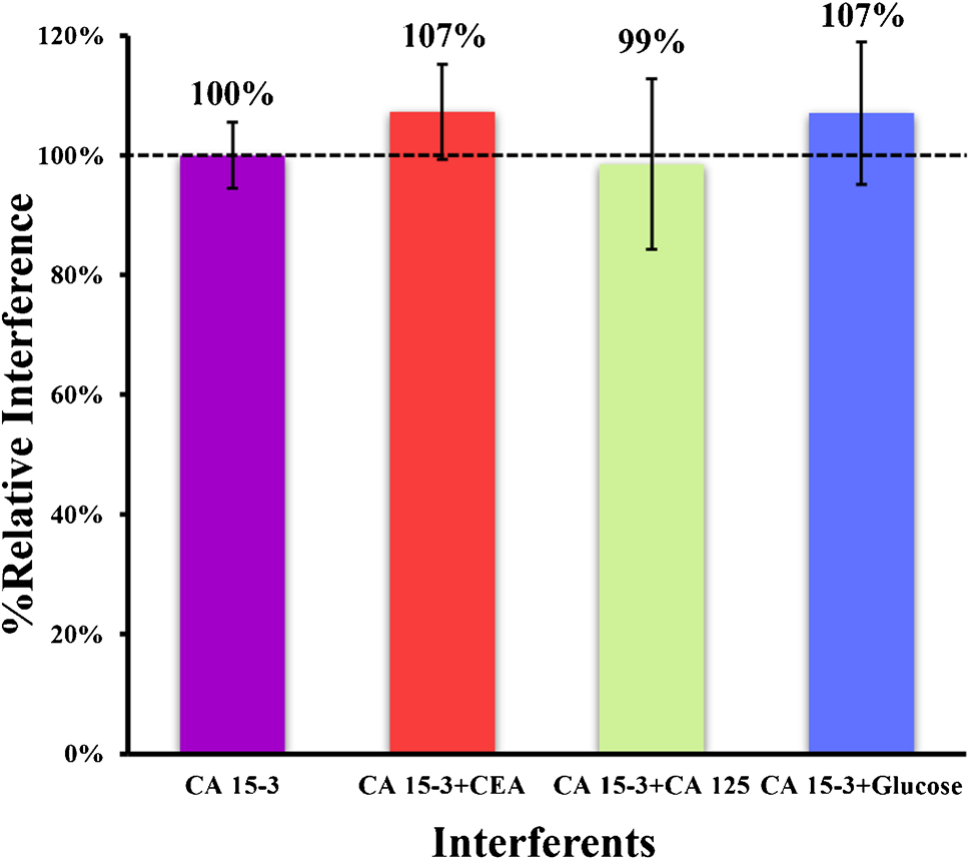
SERS signals were obtained in the selectivity study using the mixed solution method for the following possible interfering species: CEA, CA 125, and glucose

Each solution was incubated in the sensor layer for 20 min, the same time specified for the standard solutions in the calibration procedure. Overall, the results are shown in Fig. 6 and indicate low interference of CEA (7%), CA125 (1%), and glucose (7%), meaning that the sensor has affinity for the target molecule. Overall, these results show that our sensor confirms good performance in terms of specificity and selectivity.

#### Detection of CA 15–3 in Cormay serum

**Fig. 7:**
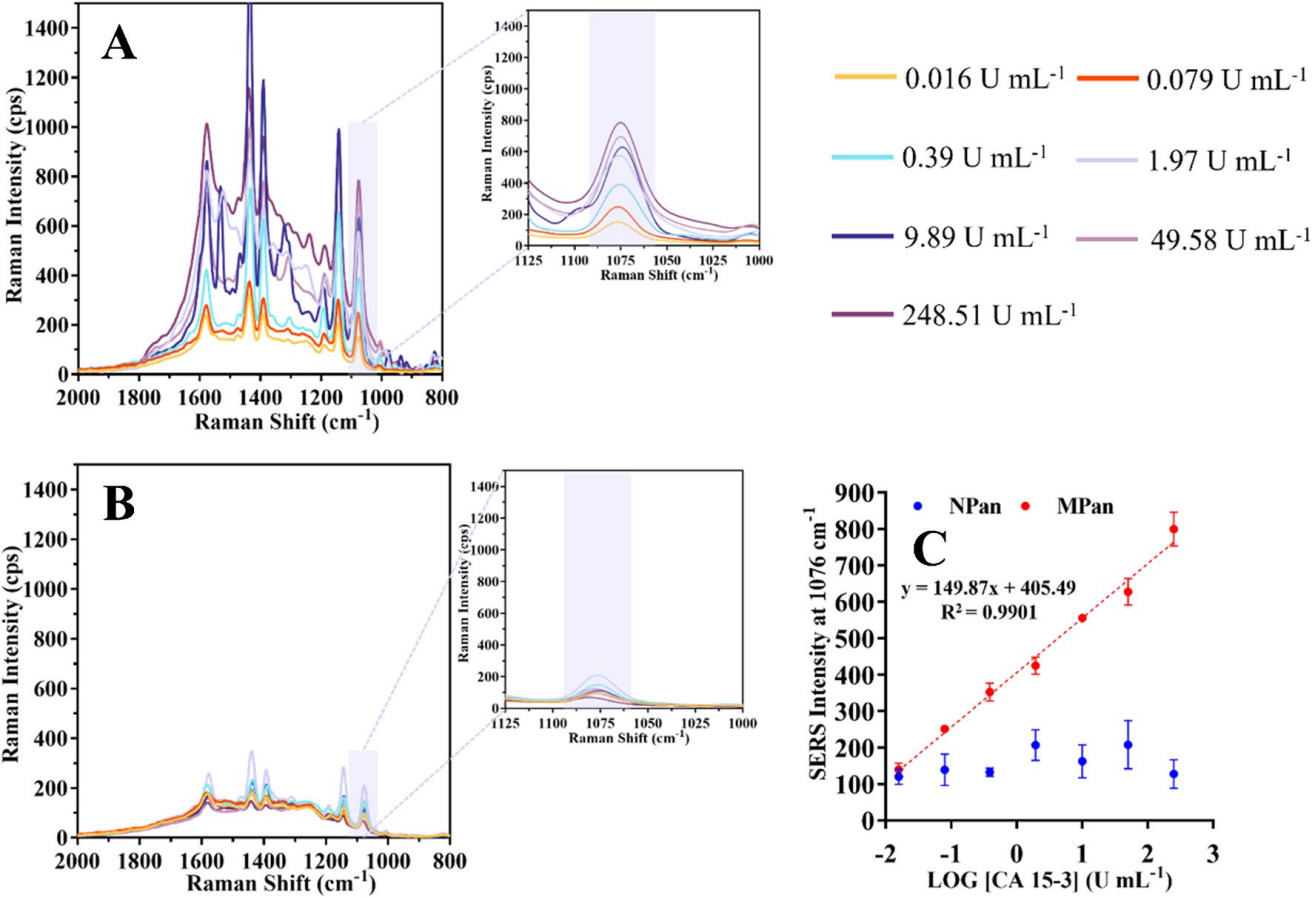
The MPan (**A**) Raman spectra and the NPan (**B**) Raman spectra after incubation in the WE with increasing concentrations of CA 15–3 in a Cormay serum solution (ranging from 0.016 to 248.51 U mL^*−*^^1^), then incubated with a SERS probe. The linear response of the sensor calibration (**C**)

Figure 7A shows the typical RAMAN spectra of the MPan and (Fig. 7B) NPan against increasing CA 15–3 concentrations prepared in Cormay serum, pH 7.4. Figure 7C shows the corresponding SERS signal calibration curve, plotting the SERS signal against the logarithm of the CA 15–3 concentration. Biosensor calibration was also performed using standard solutions prepared in a serum environment. This served the purpose of ensuring valid agreement between standards and samples. For this purpose, Cormay serum was used at 1000-fold dilution, which is very close in composition to human serum, much closer than PBS. The results obtained under these conditions for MPan and NPan are shown in Fig. 7C. In general, the serum samples showed good analytical properties and exhibited a linear range from 0.016 to 248.54 U mL^−1^, with a slope of 148.87 SERS/decade concentration and a squared correlation coefficient of 0.9901.

The NPan showed a very weak signal in the working concentration range. Compared to calibrations in the PBS buffer, the slope of the calibration increased by 15.32%, but the linear response range remained the same. In general, the sensor showed a controlled response to CA 15–3 concentration in a complex background such as the Cormay serum, indicating that it can provide accurate data when analyzing serum samples.

## Conclusions

The experimental results showed that SERS proved to be a suitable technique for monitoring the performance of the biosensor. In general, this biomimetic biosensor was created simply and shows its potential for clinical applications. It was used for the determination of CA 15–3 in real serum samples and showed a sensitive response at concentrations within the physiological range.

Although there are reports in the literature in which the detection method achieves lower values than those presented in this study, it is worth noting that the concentration of CA 15–3 in the reference serum of healthy individuals is typically less than 30 U mL^−1^, whereas much higher values are expected in cancer patients. Therefore, the sensitivity of this biosensor is more than sufficient to distinguish between cancer patients and healthy individuals. Moreover, this method is effective and provides a dual response by combining electrochemical techniques with Raman spectroscopy. A direct comparison between the two detection methods shows that SERS provides a biosensor with improved operating characteristics compared to EIS. In this context, the LLLR achieved by SERS analysis is 10 times lower than that achieved by EIS.

The current achievements lay the foundation for future improvements by extending this approach to advanced metal electrodes with Raman amplifiers, such as metal nanowire arrays and metal nanostars, which can serve as highly sensitive devices for both electrochemistry and SERS.

In summary, this device paves the way for high-precision SERS-based measurements and provides strong motivation for the development of reliable point-of-care devices shortly that contribute to the rapidly advancing field of medical applications.

### Supplementary Information

Below is the link to the electronic supplementary material.

Supplementary file1 (DOCX 1888 KB)
