# Supplementary material for: SERS biosensor with plastic antibodies for detection of a cancer biomarker protein
Source: Mikrochim Acta. 2024 Apr 4;191(5):238. doi: 10.1007/s00604-024-06327-y (PMC10991021; doi:10.1007/s00604-024-06327-y)
Supplement: Supplementary file 1 — Supplementary file1 (DOCX 1888 KB) [file 604_2024_6327_MOESM1_ESM.docx]

**Supporting Information**

**SERS biosensor with plastic antibodies for detection of a cancer biomarker protein**

*Daniela Oliveira^1^, Mariana C.C.G. Carneiro^1^, Felismina T.C. Moreira^1*^*

^1^CIETI, LabRISE, School of Engineering, Polytechnic of Porto, R. Dr. António Bernardino de Almeida, 431, 4249-015 Porto

* Felismina Moreira, School of Engineering of the Polytechnic School of Porto, R. Dr. António Bernardino de Almeida, 431, 4249-015 Porto, Portugal, Email address: [ftm@isep.ipp.pt](mailto:ftm@isep.ipp.pt)

**Table S1** – Comparison of techniques described in the literature for CA 15-3 measurement.

| Sensing Approach | Technique | Response Range U mL^-1^ | Limit of detection U mL^-1^ | Reference |
| --- | --- | --- | --- | --- |
| Antibody | Electrochemical | 1.0 to 1000 | 0.95 | [24] |
| Antibody | Electrochemical | 0.1 to 150 | 0.03 | [25] |
| Antibody | Electrochemical | 0.1 to 300 | 0.07 | [26] |
| Antibody | SERS | 0.1 to 500 | 0.13 | [27] |
| Antibody | Fluorescence | 10 to 100 | 0.027 | [28] |
| MIP | Electrochemical | 0.25 to 10 | 0.05 | [29] |
| MIP | Electrochemical | 0.10 to 100 | 0.10 | [30] |
| MIP | Electrochemical | 5 to 50 | 1.50 | [31] |
| MIP | Electrochemical | 5 to 35 | 1.16 | [32] |
| MIP | SERS | 0.016 to 248.51 | --- | This Work |


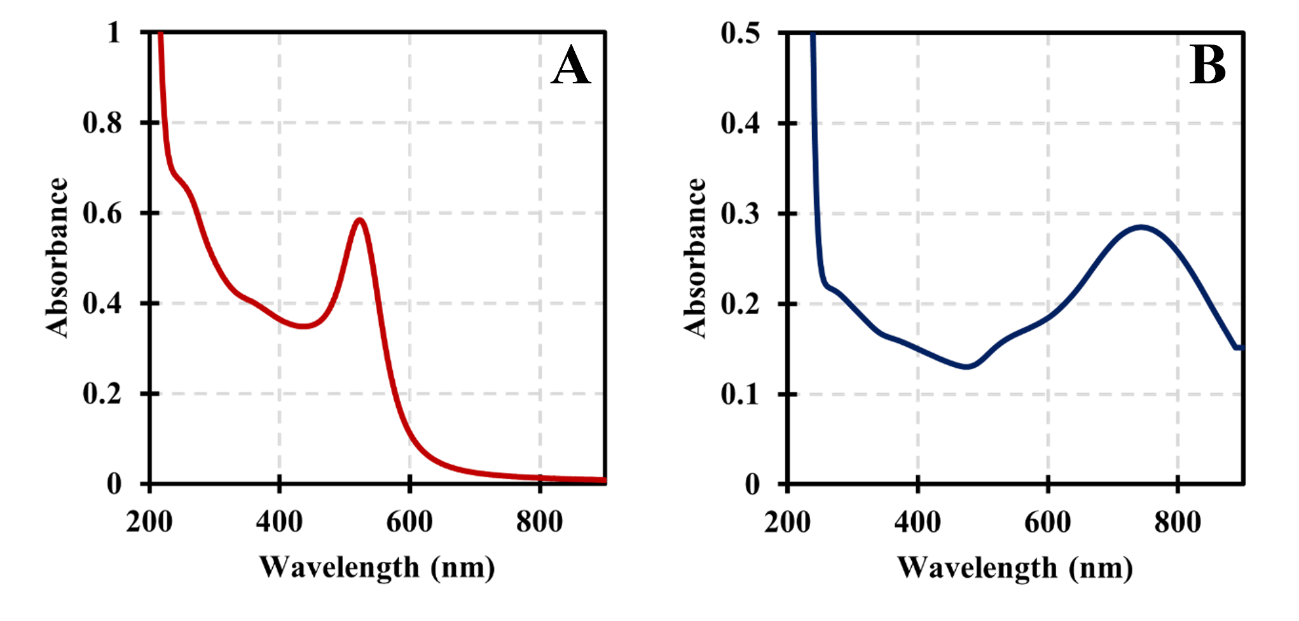


**Figure S1** – UV-Vis spectra of AuNPs+PVP and AuNSs.


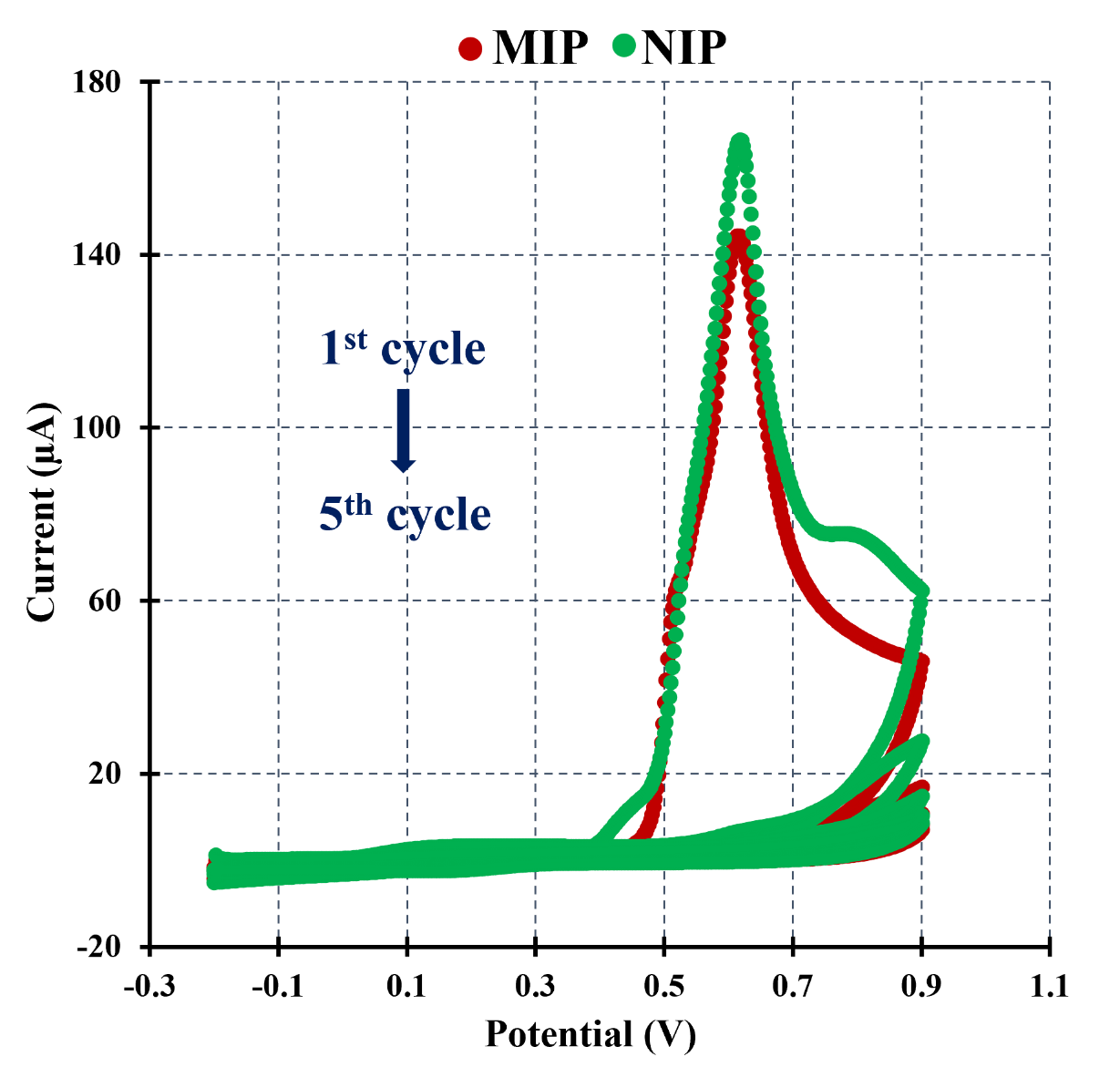


**Figure S2** – CVs of the electropolymerisation of the MPan and NPan.


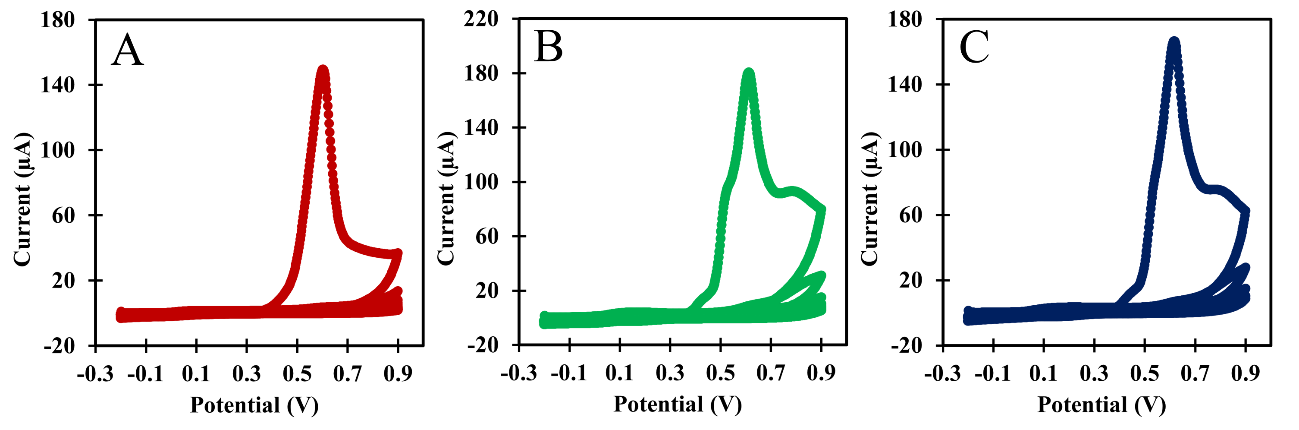


**Figure S3** – CVs of the electropolymerisation of the aniline with different number cycles. (A) 20 cycles; (B) 10 cycles; (C) 5 cycles.


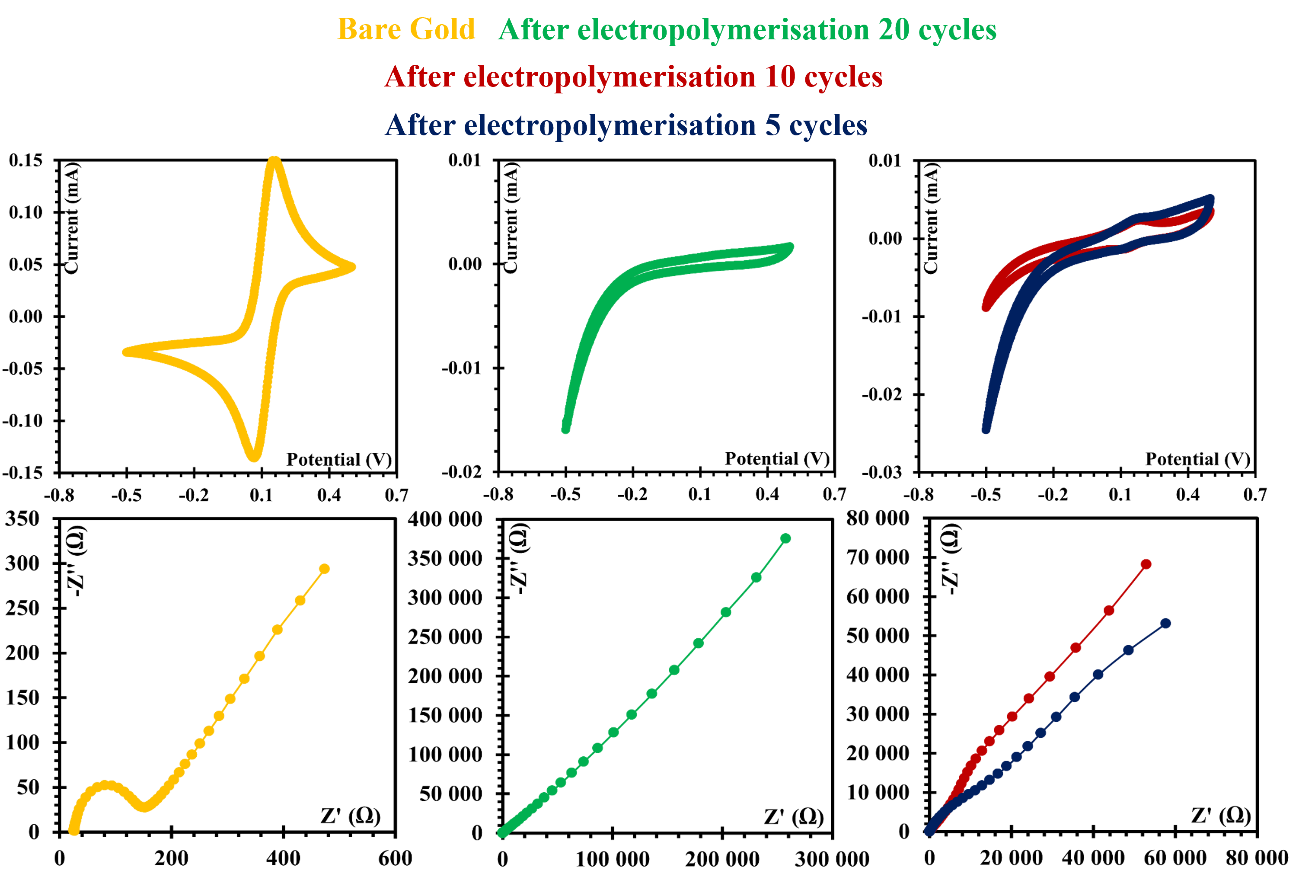


**Figure S4** – CV and EIS measurements were made after applying different number cycles during the electropolymerisation of the monomer.


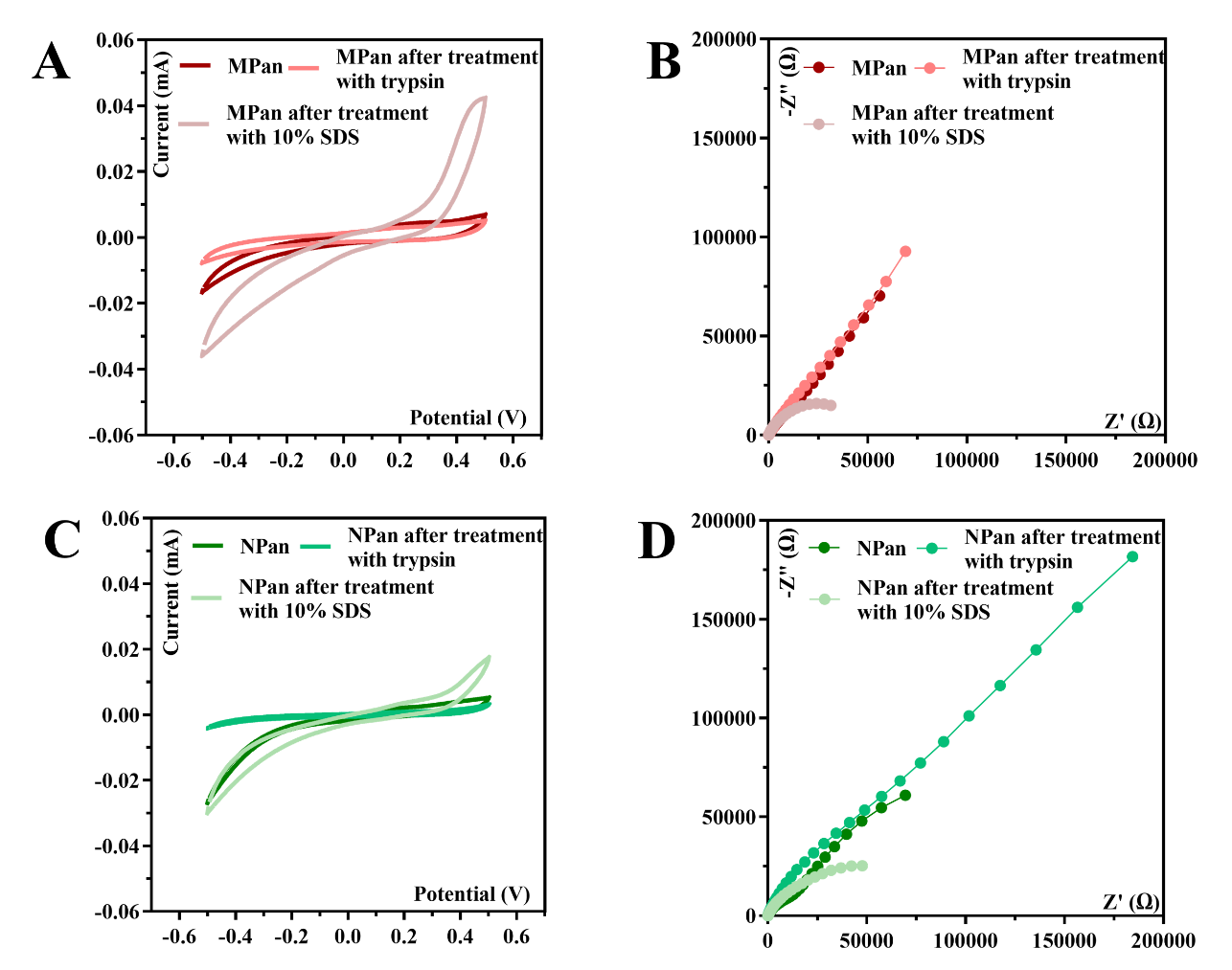


**Figure S5** – CV and EIS measurements were performed at various stages during the fabrication of MPan and NPan. The sensor's response after the removal of the target molecule is illustrated in Figures C and D for MPan and Figures E and F for NPan.


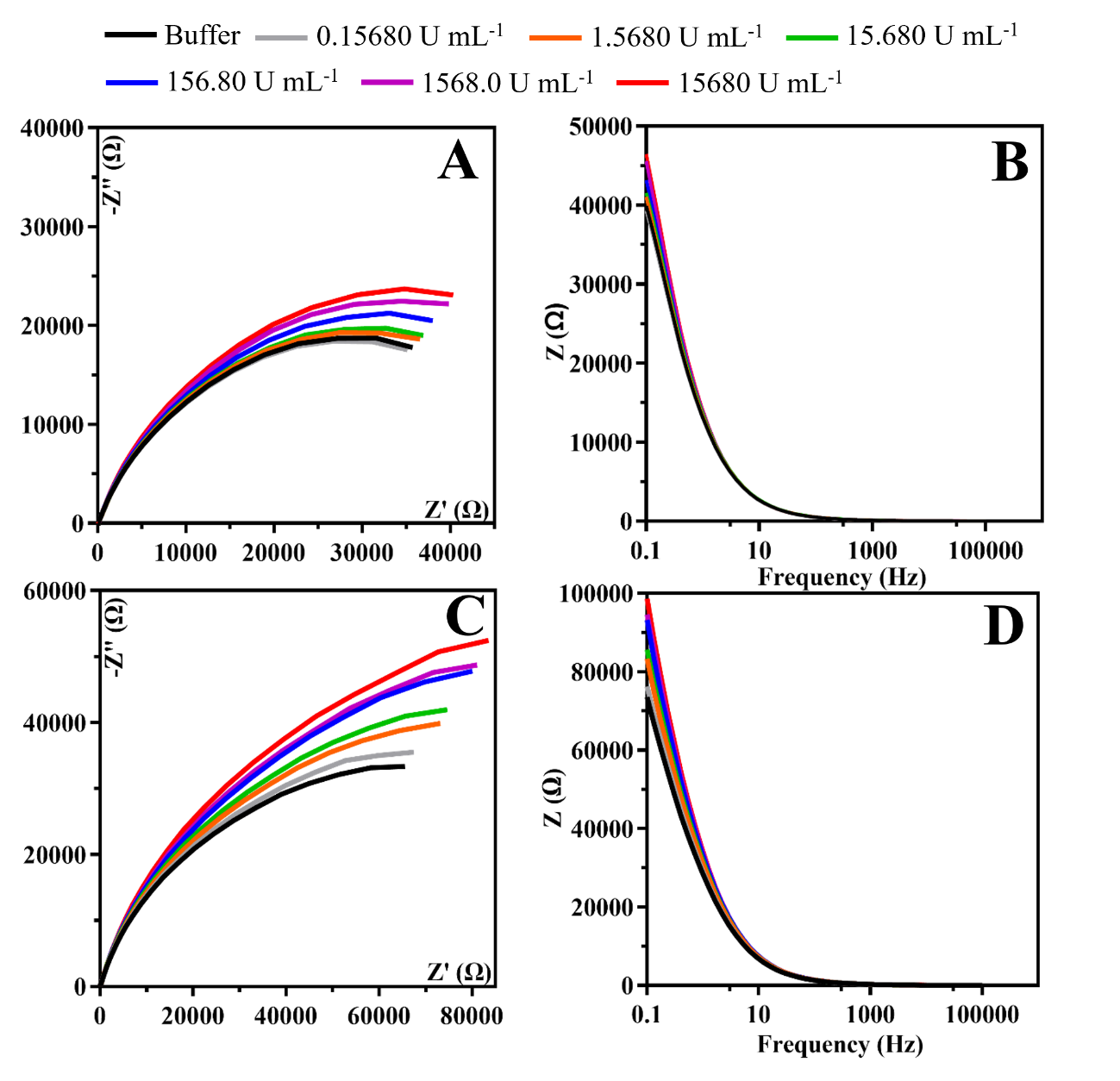


**Figure S6** – EIS (Nyquist and Bode) measurements of the last stabilization in buffer and increasing standard concentrations of CA 15-3 in MPan (A and B) and NPan (C and D).


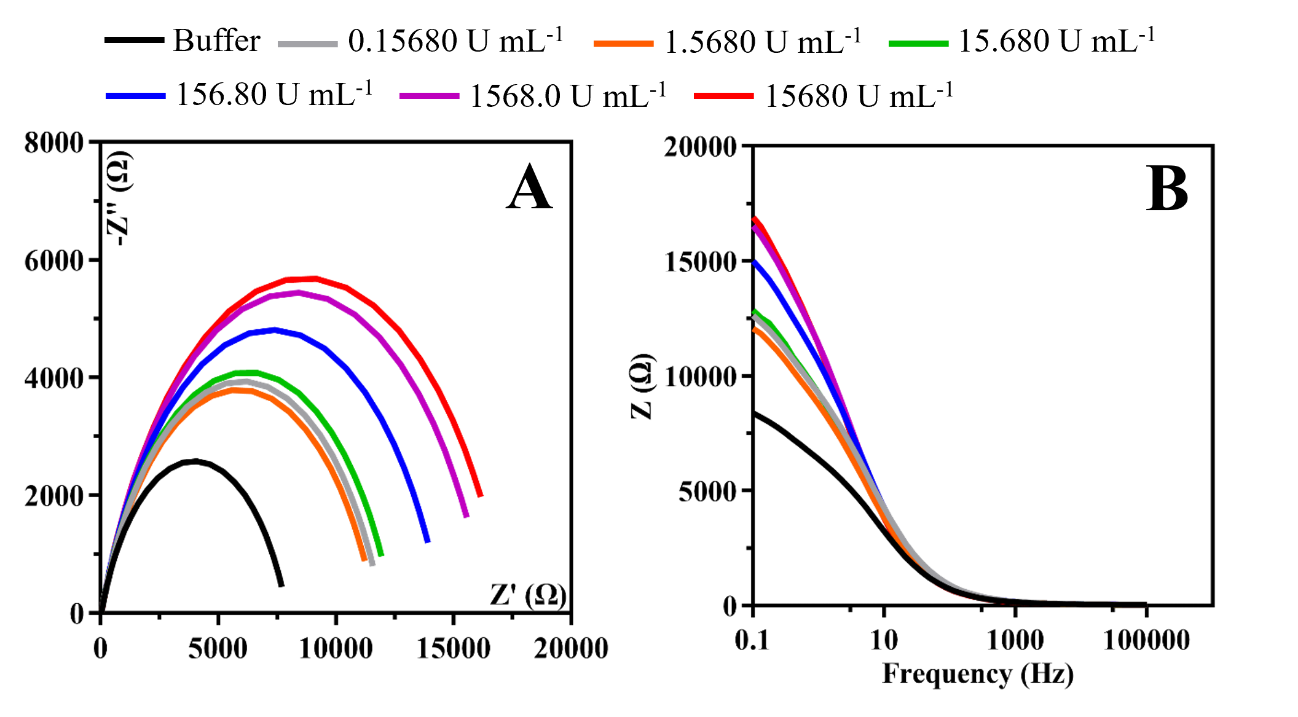


**Figure S7**– EIS (Nyquist and Bode) measurements of the last stabilization in buffer and increasing standard concentrations of CA 15-3 in NPan (A and B).
